# The Fire from Within: Multiorgan Failure with Bimodal Rhabdomyolysis from Exertional Heat Stroke

**DOI:** 10.1155/2020/1305730

**Published:** 2020-02-07

**Authors:** Onion Gerald V. Ubaldo, Khia Quiwa, Rohana Elise Rollan, Edhel Tripon, Elizabeth Sebastian

**Affiliations:** The Medical City, Ortigas Avenue, Pasig, Philippines

## Abstract

Heat stroke (HS) is a condition characterized by a rise in core body temperature and central nervous system dysfunction. It is divided into two types: classical and exertional. Exertional heat stroke (EHS) is accompanied by organ failure. Liver injury, presenting only with a rise in liver enzymes, is common but in rare conditions, acute liver failure (ALF) may ensue, leading to a potentially lethal condition. Most cases of EHS-induced ALF are managed conservatively. However, liver transplantation is considered for cases refractory to supportive treatment. Identifying patients eligible for liver transplantation in the context of an EHS-induced ALF becomes a medical dilemma since the conventional prognostic criterion may be difficult to apply, and there is paucity of literature about these specific sets of individuals. Recently, extracorporeal liver support has been gaining popularity for patients with liver failure as a bridge to liver transplant. In this case report, we present a young Filipino athlete with symptoms and clinical course consistent with EHS that developed multiorgan failure, initially considered a candidate for liver transplant and total plasma exchange, but clinically improved with supportive management alone. This patient was also found to have bimodal rhabdomyolysis during the course of his hospital stay as manifested by the bimodal rise in his creatine kinase enzymes.

## 1. Introduction

Multiorgan failure due to exertional heat stroke is an uncommon finding especially in tropical countries where inhabitants have been acclimated to the current weather. Heat stroke is a clinical entity wherein the body's core temperature rises above 40 degrees Celsius or 104 degrees Fahrenheit and is accompanied by central nervous system dysfunction [1–4]. There are two types of heat stroke: classical heat stroke (HS) and exertional heat stroke (EHS). HS is more common in warmer climates and the elderly, develops slowly over a period of days, and may be associated with neuropsychiatric complications. On the other hand, EHS is commonly seen in a younger set of individuals, associated with vigorous physical exertion, and may ultimately lead to organ dysfunction, organ failure, and death if not managed properly [2, 5, 6]. Common risk factors for EHS include illicit drug use and certain genetic conditions. Organ failure ranges from circulatory shock necessitating vasopressor use, acute renal failure (ARF) requiring renal replacement therapy (RRT), respiratory failure that may need endotracheal intubation for ventilatory support, and rarely acute liver injury (ALI) and acute liver failure (ALF) that may necessitate a discussion on liver transplantation [2, 4, 5, 7]. The survival rate of a patient with ALF with conservative medical management alone ranges from 10 to 40% [8]. In this case report, we present a case about EHS and the complications that follow this condition. Here, we present a young Filipino athlete who developed multiorgan failure with bimodal rhabdomyolysis secondary to EHS.

## 2. Case Presentation

A 27-year-old Filipino male was brought to the emergency room (ER) of The Medical City (TMC) due to loss of consciousness. The patient was brought to the ER for loss of consciousness (LOC) after running 6.8 kilometers at 34 degrees Celsius ambient temperature. He had no hydration support during training and was recovering from a recent episode of gastroenteritis. There was no note of seizure or head trauma after the incident as he was caught by his colleagues and was immediately brought to the ER. Upon assessment at the ER, his Glasgow Coma Scale (GCS) score was seven. Initial vital examination showed that he was hypotensive at 70/40 mmHg and tachycardic at 162 beats per minute in regular sinus rhythm. He had a respiratory rate of 30 breaths per minute with an oxygen saturation of 98% at room air via pulse oximetry, and his tympanic temperature was 40.2 degrees Celsius. Initial physical examination revealed he was rousable to name calling, his heart sounds were distinct, and there was no evidence of rales on posterior chest auscultation. External cooling measures with ice bags were immediately placed on his axillary regions, groin, and neck to bring down his temperature aggressively. His blood pressure was preload responsive to boluses of crystalloids given, with a blood pressure (BP) of 120/80 after volume resuscitation.

Laboratory investigations showed a creatinine level of 1.81 mg/dL with an estimated glomerular filtration rate (eGFR) of 51; blood urea nitrogen (BUN) was at 32.21 mg/dL, sodium was at 144 mmol/L, potassium was initially at 5.1 mmol/L but went down to 3.7 mmol/L after volume resuscitation, ionized calcium was at 4.6 mg/dL, venous bicarbonate was at 17 mmol/L, magnesium was at 1.62 mg/dL, random blood sugar (RBS) was at 113.2 mg/dL, creatine kinase total (CK total) was at 312 U/L, creatine kinase MM (CKMM) was at 252 U/L, creatine kinase MB (CKMB) was at 60 U/L, prothrombin time (PT) was 19.3 with an international normalized ratio (INR) at 1.61, and activated partial thromboplastin time (aPTT) was 31.0. Acid-base balance was investigated using arterial blood gas taken at room air which showed metabolic acidosis with respiratory alkalosis with a pH of 7.406, PCO_2_ 29.5, PO_2_ 84.2, HCO_3_ 18.7, and an O_2_ saturation of 96.6%. Anion gap was not computed due to the lack of chloride levels. Urinalysis showed hematuria, glucosuria, proteinuria, and bacteriuria. Urine neutrophil gelatinase-associated lipocalin (NGAL) was initially at 393 and was >6000 on repeat after 6 hours.

Radiologic investigations showed that his chest radiograph was within normal limits. Cranial computed tomography (CT) scan was done due to an episode of tonic-clonic seizures during his ER stay but eventually terminated with 5 mg intravenous diazepam. The cranial CT scan showed no structural lesions and acute pathologies such as infarcts or hemorrhages. He was then transferred to the Acute Stroke Unit (AcSU) for closer monitoring and further management.

**Figure 1:**
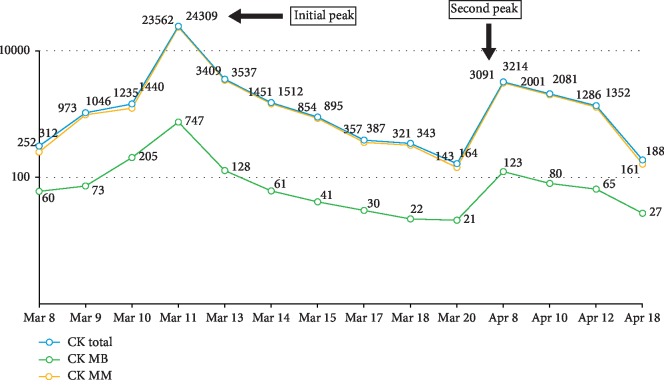
Creatine phosphokinase (CPK) trends.

**Figure 2:**
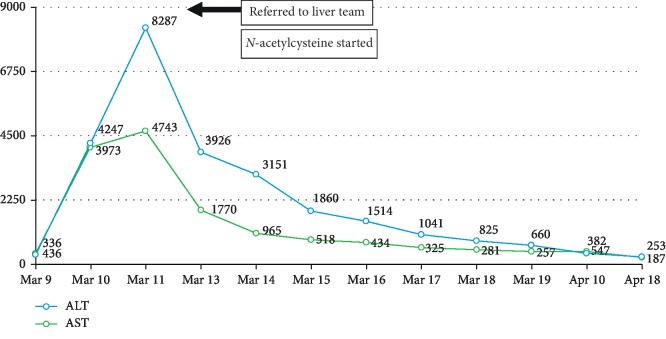
Transaminases trends.

Over the course of two days, the patient improved with supportive management of venoclysis, surface cooling measures, and antipyretics. A rectal probe was inserted to monitor his core body temperature. His urine output was adequate at 40–80 mL per hour using a foley catheter, his fever was controlled, and the creatine kinase levels were decreasing (see Figure 1). No desaturation or hypotensive episodes were noted. The AcSU team contemplated to transfer him to the regular nursing unit but on the third AcSU day, he suddenly developed hypotension as low as 70/50 mmHg, anuria, and desaturation as low as 83%. He was intubated for respiratory failure; arterial hypotension was addressed with noradrenaline and vasopressin to maintain a mean arterial pressure (MAP) of 65 mmHg to ensure sufficient organ perfusion, using serum lactate as a surrogate marker. Laboratory investigations showed persistent metabolic acidosis with oligoanuria and an increase in creatine kinase levels once more, and hence, continuous renal replacement therapy (CRRT) was initiated. His INR was noted to be elevated with decreasing platelet counts. Serum fibrinogen was requested and showed levels consistent with disseminated intravascular coagulation (DIC); hence, cryoprecipitate and fresh frozen plasma (FFP) were transfused to reverse coagulopathy. Liver transaminases were elevated, and acute liver failure was entertained (see Figure 2). He was eventually referred to a hepatologist for consult, and additional laboratory tests showed hyperbilirubinemia with low levels of albumin and phosphorus. Serial monitoring of liver transaminases, bleeding parameters, and bilirubins were done; however, the former and the latter were noted to be steadily increasing.

**Figure 3:**
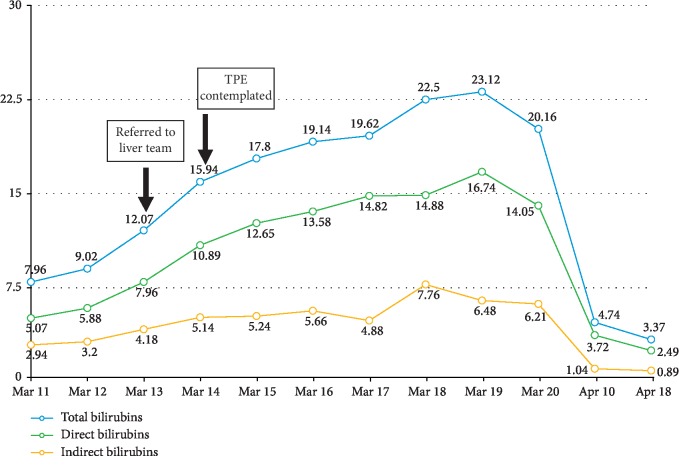
Bilirubin trends.

The patient was referred to transplant hepatology, and intravenous *N*-acetylcysteine was started at the ALF dose over 72 hours. At this time, his alanine aminotransferase (ALT) and aspartate aminotransferase (AST) were 8287 units per liter and 4743 units per liter, respectively (see Figure 2). His total bilirubin was 12.07 mg/dL, direct bilirubin was 7.96 mg/dL, and indirect bilirubin was 4.18 mg/dL (see Figure 3). Routine urine drug check was conducted showing isolated elevation of benzodiazepines in his urine, which was probably due to the premedications given during endotracheal intubation. Hepatitis titers and an autoimmune panel were checked to further investigate other etiologies of fulminant liver failure but yielded negative results. A liver ultrasound revealed fatty liver with absence of portal vein and hepatic artery thrombosis.

He was also referred to a hematologist for possible total plasma exchange (TPE) in order to temporize the ongoing hepatic injury. The decision of the team at that time was to closely monitor the laboratory trends since the patient was clinically improving. Eventually, he was liberated from vasopressors and mechanical ventilation upon correction of the metabolic acidosis. His urine output was averaging 0.5 to 1 ml per kg per hour, and he was transitioned from daily CRRT to every other day regular dialysis schedule. Physical rehabilitation was started to prevent deconditioning and muscle atrophy. His liver and hematologic profile plateaued and continued to improve; hence, plans for liver transplant and TPE were deferred. After 10 days in the AcSU, he was then transferred to the regular nursing ward.

After a couple of days at the regular nursing unit, he complained of bilateral calf pain which was initially thought to be muscle strain from physical rehabilitation. Re-examination of his creatine kinases showed a recurrence of increase in levels. This was managed conservatively with hydration and rest from physical rehabilitation. A genetics consultation was done, and carnityl palmitoyltransferase II (CPT II) levels and phenotyping were requested (pending as of this writing) to determine genetic predisposition to rhabdomyolysis as evidenced by a bimodal rise in CPKs [9]. He was eventually discharged stable and ambulatory.

## 3. Discussion

EHS is a condition which, when present, may result to multiorgan failure which includes circulatory, renal, and central nervous system disturbances, and rarely, it may cause ALF as was shown in the case [4, 6, 10]. EHS-induced ALF is a potentially lethal condition, taking into consideration the number of organ failures it is usually accompanied with [11]. In the report from the Acute Liver Failure Study Group (ALFSG), it was found that from the period of January 1988 to April 2015 in the United States, 25% percent of deaths were due to EHS-induced ALF [3].

In a report by Martinez-Infran and colleagues, they concluded that fulminant hepatic failure (FHF) is a rare result stemming from EHS, but when it does occur, which is about 90% of the time, these patients suffer from other conditions such as heart failure, severe sepsis, and respiratory failure, and is usually present in patients with significant comorbidities [12]. In the present case, we describe a 27-year-old Filipino who was in a previous good state of health and an athlete who suffered from EHS after physical exertion under unforgiving weather conditions who presented with loss of consciousness. This symptom may be explained by parameters for which we consider heat stroke, but the following events of organ failure point directly towards a diagnosis of EHS. Thus, it is important to know the circumstances leading to the chief complaint since the consideration of HS is largely based on history taking.

The initial management in HS should be geared towards reducing the core body temperature to less than 40 degrees Celsius within 30 minutes of the chief complaint, a term coined as “the golden half hour” by Inayat in his discussion of HS [1]. At present, there is a gap in the available literature regarding which method is preferred to decrease the core temperature in EHS, whether it be intravenous cooling methods via a central cooling catheter or surface cooling machines or placing ice bags into the patients groin, neck, and axilla as what was done for the patient.

Certain pathophysiological mechanisms can explain organ failure in the setting of EHS. Heat sepsis with a concomitant cytokine storm may progress to a septic shock-like state leading to vasodilation of arterioles, thus producing organ hypoperfusion [5]. With organs such as the heart, gut, and kidneys receiving less blood flow and ultimately less oxygen than usual, there is a preferential use of anaerobic metabolism to produce energy, and thus, acidosis occurs, mainly in the form of increased lactate [13].

Aside from the evident hypoperfused state contributed by heat sepsis and cytokine storm from a high core body temperature, muscle break down after physical exertion may produce pigment nephropathy in the form of elevated CPKs and rhabdomyolysis which may lead to acute renal failure. With the kidneys failing, acid-base balance remains unchecked and acidosis ensues. This vicious cycle of hypoperfusion producing acidosis leads to further vasodilatation as it promotes catecholamine receptor hyporesponsiveness [14, 15]. Most of the end-organ damage evident from EHS can be explained by the hypoperfused state which was discussed extensively above. But liver damage proceeding to liver failure is a rare occurrence in EHS especially in a young, athletic patient who was in a previous good state of health. The literature presents us with numerous theories regarding hepatic damage in the context of EHS but is mostly speculative.

In a study by Davis et al., the authors noted that the systemic inflammatory response syndrome (SIRS) is a key contributor in the multiorgan failure experienced by patients with HS, with the interleukin-1B pathway showing intimate relationship with hepatocyte damage in rats. Another hypothesis by the same authors suggested that hepatocyte damage is secondary to ischemic hepatitis from dehydration and shunting of blood from the splanchnic circulation to the skin in order to dissipate heat [3]. In another report by Martins et al., the authors referred that hepatic ischemia secondary to microthrombosis and endothelial injury may play a role in hepatic injury and failure in EHS [4]. Salathe et al. corroborate the pathophysiology presented by Davis et al., contemplating that liver failure from EHS might be a cause of splanchnic circulation being shunted to the cutaneous vessels; however, they put emphasis on release of microbial toxins from the gut that may have contributed to the sepsis-like syndrome, thus further promoting liver damage [5]. Finally, in a report by Inayat and Virk, they posited the idea that aside from heat sepsis and the cytokine storm directly contributing to the hepatocyte damage in EHS, they believe that massive hepatocyte necrosis is secondary to either extensive thermal shock or intrahepatic circulatory failure due to excessive volume resuscitation [1]. Indeed, numerous theories surround the rare occurrence of hepatic injury and failure in EHS, but the exact pathophysiological mechanism by which this occurs is still not completely understood.

Despite the gamut of processes that induce hepatocyte damage, management of EHS-induced liver injury should still follow the same dictum—organ support, avoidance of hepatotoxic medications, and close monitoring of liver function which includes bleeding parameters, clotting factors, albumin, and phosphorus levels since these patients are at risk of developing hepatic encephalopathy and disseminated intravascular coagulation, which ultimately leads to hepatic failure and carries a grave prognosis. As for the patient presented, it was apparent that he developed liver failure on the 3^rd^ hospital day in the AcSU.

The management of EHS-induced ALF is speculative as there is no present guideline available to delineate the steps for which the clinician may refer to. Most of the case series and reports reviewed showed that while EHS-induced liver injury may be asymptomatic, there may be cases of EHS-induced FHF and that majority were managed supportively and medically [2–5, 7, 16]. Supportive measures include core body temperature control to prevent heat-induced damage to tissues and organs, maintaining an adequate MAP to prevent organ hypoperfusion and preserve mitochondrial functions, adequate preload repletion in lieu of large volumes of insensible losses from a high temperature, and the presence of the mechanical ventilator. Most case reports cited above showed success in managing EHS-induced organ failure and EHS-induced hepatic failure conservatively; however, numerous case reports also show that liver transplantation may also show a role in the management of these cases. The usual indication of liver transplantation for ALF hinges on the prognostic criterion set by Clichy–Villejuif or the King's College [1, 17]. Also, ALF is one of the few conditions for which a patient can be listed as the United Network for Organ Sharing (UNOS) status 1A (urgent) in the United States (US) and “super urgent” in the United Kingdom (UK) [17]. However, application of these may not be suitable for patients who present with EHS-induced ALF/FHF [10], and to date, there still seems to be a paucity of literature regarding indications for liver transplantation in this context.

Several factors with the patient that are included in the King's college and Clichy criteria for prognostication may be misconstrued in the setting of EHS. First, impaired consciousness may be a result prolonged hyperthermia and acidosis rather than hepatic encephalopathy. Second, the rise in liver transaminases may be an overestimation due to the parallel rise in CPKs while the patient was having rhabdomyolysis. Third, due to the heat-induced vascular injury, the coagulation tests may also be overestimated. Hence, there is a difficulty in applying the same set of criteria for liver transplantation in ALF patients to those with EHS-induced ALF [8]. All of these factors present a medical dilemma for patients with EHS-induced ALF contemplating to undergo liver transplantation.

Ichai and colleagues in a recent multicenter study done in 2018 retrospectively profiled patients between 1995 and 2016 with a diagnosis of EHS-induced severe acute liver injury (sALI) defined as PT < 50% at admission or during hospitalization or ALF defined by PT < 50% and hepatic encephalopathy. A total of 26 patients were included with nine (9) patients (37.5%) deemed candidates for liver transplantation based on either the King's college criteria or the Clichy–Villejuif criteria. Out of the 9 patients, four (4) (44.5%) underwent liver transplantation. The remaining five (5) patients were managed conservatively. The overall survival rates of patients without transplantation and with transplantation were 100% and 75%, respectively signifying that most patients with EHS-induced sALI or ALF can be managed conservatively even if they fulfill a certain criteria for transplantation [10].

Martins et al. conveyed a PubMed search of English literature using the keywords liver, transplant, and heat stroke. They gathered a total of 27 studies with more than half reporting spontaneous recovery of hepatic function with conservative management alone [4]. Upon conduction of our own PubMed search of English literature using the same keywords but limiting the reports exclusive to patients who underwent liver transplant for EHS, we generated a total result of 32 studies with 11 studies reporting liver transplantation from EHS [8, 11, 12, 18–20]. A total of 13 liver transplant patients were included in this search (from the 11 studies). Out of the 13 patients, 3 expired either to intractable liver failure despite transplantation or chronic rejection [19, 20]. We found out that most of the indications for transplant for EHS-induced ALF were due to lack of response to supportive and medical therapy, using liver transplant as a method mainly for rescue therapy.

**Figure 4:**
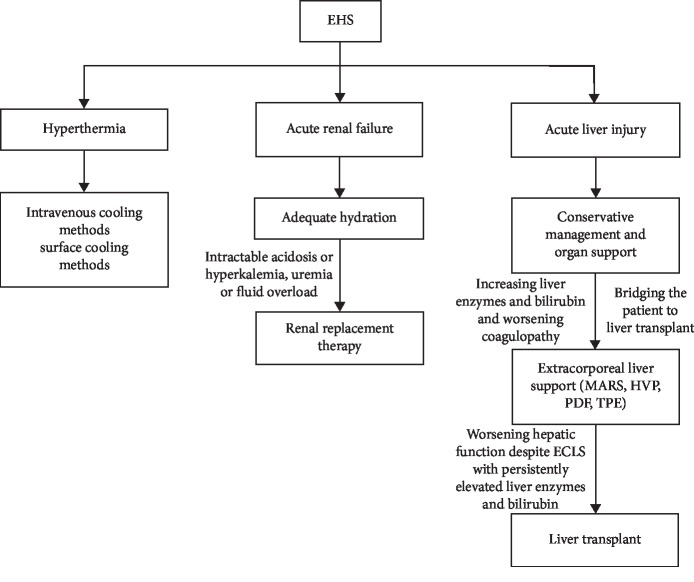
Algorithm for management of organ failure in exertional heat stroke (EHS). Molecular Adsorbent Recirculating System (MARS), high-volume plasma exchange (HVP), plasmadiafiltration (PDF), and total plasma exchange (TPE) are examples of extracorporeal liver support (ECLS).

As part of medical treatment sans liver transplant, the Molecular Adsorbent Recirculating System (MARS), high volume plasma exchange (HVP), plasmadiafiltration (PDF), and total plasma exchange (TPE) are some of the extracorporeal measures that have shown increased usage in the previous years. These modalities act as bridges for the patient to temporize the ongoing liver failure while awaiting definitive management. In isolated case reports, extracorporeal measures such as PDF have shown to improve outcomes by removing cytokines from patients suffering from heat stroke [21]. Larger clinical trials were conducted but still lacked enough evidence for extracorporeal measures to be part of the routine standard of care for ALF patients [22, 23]. Further investigations are still necessary to supply evidence for routine use of these methods and the timing of initiation as well, especially in the context of EHS-induced ALF.  Figure 4 demonstrates a simplified algorithm on the management of EHS-induced organ dysfunction. For our patient, we considered using extracorporeal means (TPE) during the time when liver transplantation was being contemplated. This was due to the continuous rise of transaminases and bilirubins. However, the patient clinically improved despite deteriorating biochemical values which later on plateaued. The patient continued to improve clinically and biochemically, and the TPE was deferred.

Finally, our patient presented with a bimodal rise in CPKs, which was initially thought to be secondary to intensification of his physical rehabilitation program. However, on literature search, a report by Yoshizawa et al. found interesting findings regarding bimodal elevations in CPKs in the Japanese population. They found six (6) studies reporting bimodal rhabdomyolysis after heat stroke, all of which had Japanese patients. It was not discounted that intensifying physical rehabilitation could possibly be a cause of the bimodality due to reinjury of collapsed muscles during the acute phase of heat stroke. But a genetic predisposition was also entertained, specifically the CPT II enzyme which is responsible for energy production during prolonged fasting or exercise. The authors noted that a thermolabile genetic phenotype of CPT II has been described in China and Japan. This genetic variant may cause the bimodality of rhabdomyolysis during even the mildest of rehabilitation as seen in the Japanese population [9]. Accordingly, our patient has a Japanese lineage and if proven to have this genetic variant, it may have repercussions of him being an athlete in the future. Due to limitations in finances, further genetic work up for the patient was postponed for a later date.

To conclude, EHS is an under-recognized condition that causes multiorgan failure and is a potentially lethal condition especially if accompanied by EHS-induced ALF [3]. A high index of suspicion based on clinical course and good history taking is paramount. Conservative management is certainly an option for patients with EHS-induced ALF with clinical and biochemical improvement occurring for the majority. Supportive management includes correction of the hypoperfused state and acid-base disorders, avoidance of medications that may potentiate hepatocyte damage, and judicious correction of insensible losses guided with the concepts of preload responsiveness. Clinicians should also be aware of the option of liver transplantation and activating the institution's liver team if circumstances are refractory to supportive management. The clinician should also be wary that the conventional prognostic criteria for transplantation may not be applicable for patients with EHS-induced ALI and that extracorporeal liver support methods (MARS, HVP, and TPE) still need further investigations to supply evidence before routine use can be recommended. Finally, Asians may have a genetic predisposition to have the thermolabile genetic variant of CPT II, which may cause bimodality of rhabdomyolysis, as may be the case of our patient. Further research on a specific prognostic transplant criterion is recommended specifically for EHS-induced ALF as well as studies on genetic predisposition that may aid in the management and prognostication in a similar subset of patients.
